# Amide proton transfer weighted imaging combined with dynamic contrast-enhanced MRI in predicting lymphovascular space invasion and deep stromal invasion of IB1-IIA1 cervical cancer

**DOI:** 10.3389/fonc.2022.916846

**Published:** 2022-09-12

**Authors:** Qingling Song, Shifeng Tian, Changjun Ma, Xing Meng, Lihua Chen, Nan Wang, Liangjie Lin, Jiazheng Wang, Qingwei Song, Ailian Liu

**Affiliations:** ^1^ Department of Radiology, First Affiliated Hospital, Dalian Medical University, Dalian, China; ^2^ Department of Radiology, Dalian Women and Children’s Medical Group, Dalian, China; ^3^ Clinical & Technical Support, Philips Healthcare, Beijing, China

**Keywords:** cervical cancer, amide proton transfer, dynamic contrast-enhanced, MRI, lymphovascular space invasion, deep stromal invasion

## Abstract

**Objectives:**

To investigate the value of amide proton transfer weighted (APTw) imaging combined with dynamic contrast-enhanced magnetic resonance imaging (DCE-MRI) in predicting intermediate-risk factors of deep stromal invasion (DSI) and lymphovascular vascular space invasion (LVSI) in cervical cancer.

**Methods:**

Seventy patients with cervical cancer who underwent MRI before operation from July 2019 to February 2022 were retrospectively included in this study. Clinical information including age, histologic subtype etc. were recorded for patients. ATPw imaging parameter APT_mean_ and DCE-MRI parameters K^trans^, K_ep_ and V_e_ were measured and analyzed. The independent-sample t-test, Mann-Whitney U test, or Chi-square test was used to compare the differences of parameters between DSI/LVSI positive and negative groups. Logistic analysis was used to develop a combined predictive model. The receiver operating characteristic curve was for predictive performance. ANOVA and Kruskal-Wallis test were used to compare the differences of consecutive parameters among multiple groups.

**Results:**

K^trans^ and SCC-Ag were independent factors in predicting DSI; K^trans^+SCC-Ag had the highest AUC 0.819 with sensitivity and specificity of 71.74% and 91.67%, respectively. APT_mean_ and K^trans^ were independent factors in predicting LVSI; APT_mean_+K^trans^ had the highest AUC 0.874 with sensitivity and specificity of 92.86% and 75.00%, respectively. K^trans^ and Ve could discriminate coexistence of DSI and LVSI from presence of single one, APT_mean_ could discriminate the presence of DSI or LVSI from no risk factor presence.

**Conclusion:**

The combination of APTw and DCE-MRI is valuable in predicting intermediate-risk factors of DSI and LVSI in cervical cancer.

## Introduction

Cervical cancer is the fourth most common female malignancy worldwide. Its incidence is particularly high in developing countries; in 2020, the highest mortality rate (approx. 342,000 deaths) occurred in low- and middle-income countries (1, 2). Deep stromal invasion (DSI), lymphovascular space invasion (LVSI), and tumor size larger than 4 cm are the risk factors for cervical cancer recurrence (3). Although DSI and LVSI have not been included as the staging criteria in the revised 2018 International Federation of Gynecology and Obstetrics (FIGO) staging system (latest revision) (4), their correlation with treatment and prognosis has received extensive clinical attention. Adjuvant therapy is usually recommended if the co-occurrence of DSI and LVSI are found after the operation according to the Sedlis criteria (5). However, previous studies showed that single IFR still was associated with worse outcome. LVSI is an important step in lymph node metastasis, and patients with LVSI have a significantly worse prognosis (6–8). DSI was associated with not only lymph node metastasis but also parametrial involvement, that was also related to poor prognosis (9–11). Though neoadjuvant chemotherapy was not recommended to early-stage disease, it was able to decrease the adverse pathological findings (12). Thus, it might be beneficial for patients to identify DSI or/and LVSI preoperatively and to receive intervention timely.

Studies have shown that patients with multiple intermediate-risk factors (IFRs) have a worse prognosis than patients without or with one single IFR (13), thus, Sedlis criteria was adopted by many centers since it was released. Postoperative pathological examination can confirm the DSI/LVSI status, but its lag in diagnosis would make DSI/LVSI positive patients receive dual treatment. For decreasing the adverse effects of dual treatment, ESGO/ESTRO/ESP guidelines suggested that chemoradiotherapy and brachytherapy are more suitable for the patients who intended to receive both surgery and adjuvant therapies (14). The biopsy of conization can be used as a preoperative method for accurate pathological diagnosis of DSI or/and LVSI status, and thus help the selection of treatment for early-stage cervical cancer patients (15). However, as an invasive approach, it may also lead to excessive bleeding and inflammatory reaction in the local cervical tissue after surgery. Therefore, a non-invasive assessment of the coexistence of DSI and LVSI before initial treatment would also be of great value for cervical cancer patients in defining patient therapeutic strategies and evaluating prognosis or tailor follow-up intervals (16).

Magnetic resonance imaging (MRI) offers a way for high soft-tissue resolution, multi-directional and multi-contrast medical imaging. Conventional MRI can locate tumor and clearly show the relationship between cervical lesions and adjacent structures, but it identifies malignancy mainly based on the abnormal signal intensity and morphology. More and more emerging functional MRI methods provide valuable information to assess the tumor biological behaviors. Amide proton transfer weighted (APTw) imaging is an emerging contrast media-free molecular MRI technique that detects endogenous mobile peptides and proteins in tissue *via* chemical exchange saturation transfer (CEST), thus resulting in increased APTw signal intensity values in malignancy (17, 18). So far, APTw has been used to assess the differentiation of squamous cell carcinoma from adenocarcinoma and tumor grading in cervical cancer (19–21).

Dynamic contrast-enhanced MRI (DCE-MRI) is another useful tool that quantitatively analyzes tissue microcirculation perfusion state and vascular permeability through the pharmacokinetic model and quantitative parameters (22). DCE-MRI is used to evaluate LVSI, lymph node metastasis, and other pathological features of cervical cancer (23). Considering that APTw can reflect the growth of tumor cells by detecting endogenous proteins, while DCE-MRI is capable of reflecting tumor vascularity, the combination of the two methods may improve medical imaging-based evaluation of DSI and LVSI and benefit the subsequent treatment planning.

The purpose of this study was to explore the value of APTw combined with DCE-MRI in preoperative prediction of LVSI or/and DSI in stage IB1-IIA1 cervical cancer.

## Materials and methods

### Patients

Patients with biopsy-proven cervical cancer who underwent MRI examination from July 2019 to February 2022 in our hospital were retrospectively included in this study. Inclusion criteria were: (1) patients diagnosed with stage IB1-IIA1 (except IB3) squamous cell carcinoma and adenocarcinoma, according to 2018 FIGO staging system  (4); (2) patient age ranging from 18 to 75 years old; (3) MRI examination included APTw and DCE-MRI sequences performed 2 weeks before surgery; (4) patient who underwent radical hysterectomy with pelvic lymphadenectomy. Exclusion criteria were: (1) did not undergo surgical treatment in our hospital; (2) those who received treatment for cervical lesions before surgery; (3) pathological data regarding LVSI and DSI were incomplete; (4) lesions were not clearly visible on images, or artifacts affected the observation and measurement of the lesion; (5) those with other malignant tumors.

**Figure 1:**
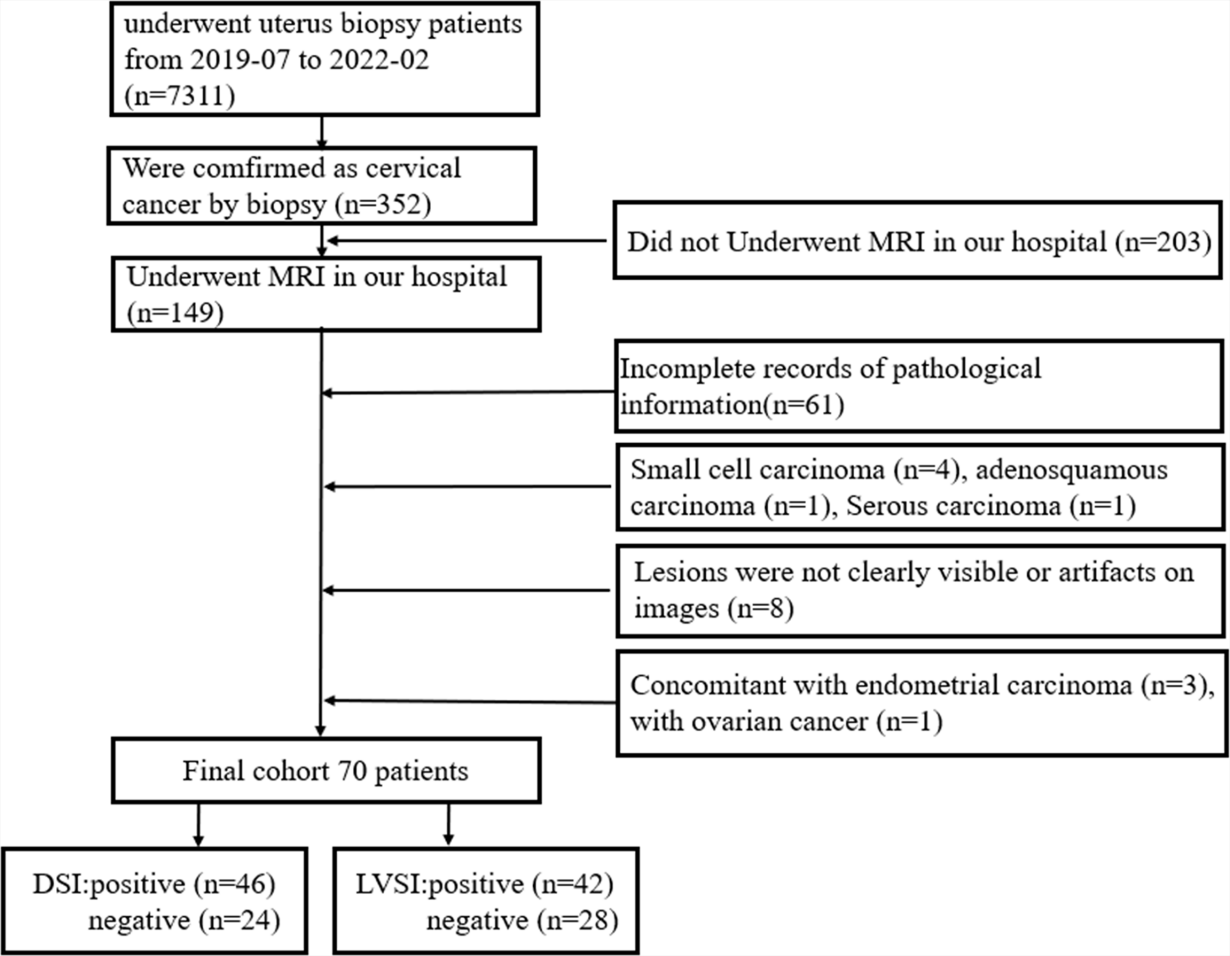
The study flow diagram, including the patient selection process.

Three hundred fifty-two patients were confirmed as cervical cancer by biopsy. Among them, 282 were excluded due to incomplete MRI scans, incomplete pathological information, image artifacts, cancer types beyond squamous cell carcinoma and adenocarcinoma, or concomitant malignancies. A total of 70 patients were finally included in this study. The study flow chart is shown in **Figure 1**. The hospital review board approved this study (approval number PJ-KS-KY-2021-180) and waived the need for informed consent.

Previous studies have shown that the depth of stromal invasion >1/2 can clearly affect the prognosis (24). Therefore, based on the above findings, patients were divided into DSI positive group (invasion depth >1/2, 46 cases) and DSI negative group (invasion depth ≤1/2, 24 cases). In addition, patients were divided into LVSI positive group (42 cases) and LVSI negative group (28 cases) according to the histopathological examination. Finally, patients were further divided into 3 groups: group 1: DSI and LVSI were both negative (11 cases); group 2, only DSI or LVSI was positive (30 cases); group 3: both DSI and LVSI were positive (29 cases).

The mean age of the patients was 54.9 ± 10.0 years (range from 32-68 years). In addition, 58 cases had vaginal bleeding symptoms, 12 cases had no vaginal bleeding symptoms; 27 women were premenopausal, 43 cases were postmenopausal; 46 cases were stage IB, and 24 cases were stage IIA disease. The preoperative SCC-Ag value (squamous cell carcinoma antigen) was 2.52 (1.46, 5.74) μg/L. There were 50 cases of squamous cell carcinoma and 20 cases of adenocarcinoma, 45 cases with poor differentiation, and 25 cases with high-moderate differentiation (**Table 1**).

**Table 1:** Comparison of clinical characteristics and pathological features between DSI and LVSI positive and negative groups.

Parameters	Positive	Negative	*P*
	DSI-positive (n=46)	DSI-negative (n=24)	
Age	56.2 ± 10.2	52.5 ± 9.5	0.137
FIGO stage			0.347
IB	32	14	
IIA	14	10	
Menstruation			0.367
Premenopausal	16	11	
Menopause	30	13	
Symptom			1.000
Vaginal bleeding	38	20	
No bleeding	8	4	
Histologic subtype			0.937
Squamous cell carcinoma	33	17	
Adenocarcinoma	13	7	
Differentiation degree			0.072
Poor differentiation	33	12	
Well-moderate differentiation	13	12	
SCC-Ag	3.94 (2.03, 6.72)	1.78 (1.01, 2.56)	**0.002**
	**LVSI-positive** **(n=42)**	**LVSI-negative** **(n=28)**	
Age	53.2 ± 11.2	56.3 ± 9.6	0.252
FIGO stage			0.758
IB	27	19	
IIA	15	9	
Menstruation			0. 920
Premenopausal	16	11	
Menopause	26	17	
Symptom			0.524
Vaginal bleeding	36	22	
No bleeding	6	6	
Histologic subtype			0.105
Squamous cell carcinoma	33	17	
Adenocarcinoma	9	11	
Differentiation degree			0.309
Poor differentiation	25	20	
Well-moderate differentiation	17	8	
SCC-Ag	3.54 (1.88, 5.89)	2.09 (0.74, 3.82)	**0.015**

The bold values provided in Tables mean the differences were statistically significant.

### MRI protocol

Pelvic scans were performed using a 3.0T MR scanner (Ingenia CX, Philips Healthcare, Best, the Netherlands) with a 32-channel body coil. All patients fasted 12h before the examination and were not allowed to drink 4 hours before the exam. MR sequences included T2 weighted imaging (T2W), APTw imaging, and DCE-MRI (40 phases). Before the DCE-MRI scan, two different flip angles (12° and 15°) were used for T1 mapping scans. After the first stage, the contrast agent gadopentetate dimeglumine (Gd-DTPA, Beijing Beilu Pharmaceutical Co., Ltd., Beijing, China) were injected at a rate of 2.5ml/s and a dose of 0.2ml/kg. Images were then collected at 39 consecutive periods, each period of 6.6s. The detail scan parameters are shown in **Table 2**.

**Table 2:** The parameters of MRI scans.

	T_2_W	APTw	DCE-MRI
Plane direction	Sagittal	Sagittal	Sagittal
TR(ms)/TE (ms)	4520/84	6400/8	3.8/1.8
FOV (mm^3^)	250×250×99	130×130×49	250×250×84
Plane Resolution	0.95×0.95	2.0×2.0	1.4×1.8
Matrix	264×264	64×65	180×140
Flip angle	90	90	8
Slice Thickness/SliceGap (mm)	4.0/1.0	7.0/0	3.5/0
Scan time	1min58s	5min3s	4min5s

APT-weighted imaging was scanned with a 3D fast spin-echo sequence, and fat suppression was performed using a chemical shift frequency selection method. Data from 7 saturation frequency points ( ± 2.7, ± 3.5, ± 4.3, and -1540 ppm) were measured, and the final APT image was obtained by signal fitting and B0 field correction. Under continuous RF irradiation at a power of 2.0 µT, the saturation time for amide protons lasted 2 s. The B0 field map was calculated by 3 repeated acquisitions at the +3.5 ppm saturation frequency with different echo times. The APT value was obtained by calculating the asymmetry of the traditional magnetization transfer effect at 3.5 ppm on both sides of the water signal:


$$APT(\%)=MTRasym(3.5ppm)\times 100\%=(S_{sat}(-3.5ppm)/S_{0}-S_{sat}(+3.5ppm)/S_{0}))\times 100\%$$

where S0 is the water signal strength at a saturation frequency of 1540 ppm, and S_sat_ is the water signal strength at a saturation frequency of +3.5/-3.5 ppm after B0 correction.

### Image analysis

Image analysis was performed independently by two radiologists (with 5- and 3-years’ experience in pelvic MRI interpretation, respectively) who were blinded to DSI and LVSI status. The APT and DCE-MRI images were reconstructed and transmitted to the workstation (IntelliSpace Portal, ISP v9.0, Philips Healthcare) for the subsequent analysis.

### ROI delineation for APTw imaging and DCE-MRI

**Figure 2:**
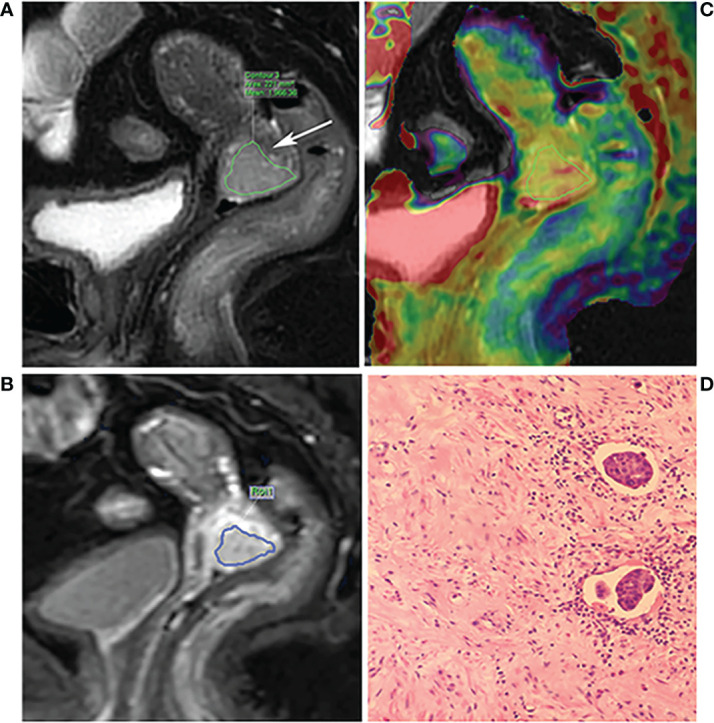
A 68-year-old patient with poor differentiation squamous cell cervical cancer with LVSI and DSI. **(A)** The T2WI and the arrow show the lesion. **(B)** The fusion image of APT pseudo color image and T2WI. The ROI was manually placed along the edge of the largest area of the tumor by referring to the T2WI, avoiding the necrotic area. **(C)** The DCE-MRI image; the ROI was manually placed by referring to the T2WI. **(D)** Hematoxylin-eosin (×200) showing LVSI positive.

First, the largest layer of the tumor was selected on sagittal T2WI. The regions of interest (ROI) were then delineated along the contour of the solid tumor part (saved about 1-2 mm along the edge of the lesion) using the freehand function (**Figure 2A**). The APTw image was then fused with the sagittal T2WI. The largest layer of the tumor was also selected on the fused image. Referring to the above method, the ROI of the tumor on APTw image was delineated on the fused image by keeping consistency between the ROIs of T2WI and APTw images. Finally, the APT_mean_ was recorded, as shown in **Figure 2B**. Referring to the ROI of T2WI, the ROI at the largest slice of DCE-MRI was selected (**Figure 2C**), after which the DCE-MRI parameters, values for the volume transfer constant (K^trans^), the rate constant of backflux (K_ep_), and the volume fraction of extravascular extracellular space (V_e_) were recorded. Parts including necrotic cysts, hemorrhage, and mucus were avoided in all ROIs.

### Histopathological evaluation

All tissues were routinely histopathological examined after operation. Briefly, tissues were embedded in paraffin, stained with hematoxylin-eosin, and examined microscopically. The lymphovascular space invasion and stromal invasion depth were recorded. If tumor cells were visible in spaces surrounded by endothelial cells without muscle wall structures, the patient was classified as LVSI positive (**Figure 2D**); and patients without LVSI observed in the whole field were classified as LVSI negative. The depth of tumor invasion into the stroma was determined by measuring the length of the tumor from the cervical surface. According to the fraction of tumor invading the cervical wall thickness, depth of stromal invasion was classified as inner third and outer third, while middle third invasion was divided into less than half which was DSI negative or more than half which was DSI positive.

### Statistical analysis

Statistical analysis was performed using SPSS 21.0. Intra-group correlation coefficient (ICC) was used to test the consistency of image parameters APT_mean_, K^trans^, K_ep_, V_e_ measured by two radiologists, and the data with ICC>0.75 were averaged. The Kolmogorov-Smirnov test was used to analyze whether the variables were normally distributed. The data with normal distribution was tested by independent sample t-test, and the data that did not conform to normal distribution was tested by Mann-Whitney U test to compare the differences of parameters between DSI, LVSI positive, and negative groups. Chi-square or Fisher’s exact test was used to compare the differences of categorical variables, such as vaginal bleeding, menstrual status, tumor stage and type, degree of differentiation between DSI, LVSI positive, and negative groups. The parameters with differences between DSI and LVSI positive and negative groups were included in binary logistic regression analysis to establish combined parameters for predicting DSI and LVSI. Receiver operating characteristic curve (ROC) was used to analyze the performance of the above parameters in predicting DSI and LVSI, including the area under curve (AUC), sensitivity, and specificity. Delong’s test was used to analyze whether there was a difference in predictive power between univariate variables and combined parameters that differed between DSI and LVSI positive and negative groups. In the analysis of the coexistence of DSI/LVSI, the one-way ANOVA test was applied to analyze the differences between normally distributed data; the Kruskal-Wallis test to analyze the differences between non-normally distributed data; the Chi-square or Fisher’s exact test to evaluate the differences in categorical variables. A *P* value< 0.05 was considered statistically significant.

## Results

### Agreement between two observers

The ICCs of APT_mean_, K^trans^, K_ep_, and V_e_ measured by two observers were all >0.75, and the average values of the two measurement parameters were taken for subsequent analysis, as shown in **Table S1**.

### Comparison of clinicopathological characteristics between DSI or LVSI positive and DSI or LVSI negative groups

The SCC-Ag was higher in the DSI-positive group compared to the DSI-negative group (*P*=0.002). In addition, there was no significant difference in age, FIGO stage, menstrual status, symptoms, histological type, and degree of differentiation between the DSI-positive group and DSI-negative group (all *P*>0.05). SCC-Ag was also higher in the LVSI-positive than that in LVSI-negative group (*P*=0.015). While there was no significant difference in other clinicopathological features between two groups (all *P*>0.05). The detailed clinicopathological results are shown in **Table 1**.

### Comparison of imaging parameters between DSI or LVSI positive and DSI or LVSI negative groups

The K^trans^ and V_e_ values were significantly higher in the DSI-positive group compared to the DSI-negative group (both *P*<0.05), while there were no statistically significant differences in APT_mean_ and K_ep_ between the two groups (both *P*>0.05).

**Figure 3:**
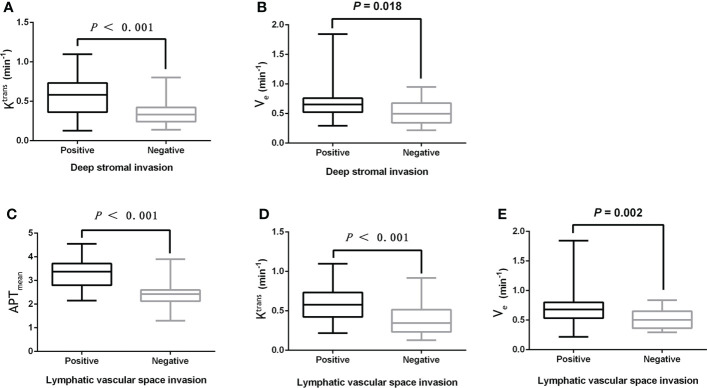
**(A, B)** The boxplot showing the difference of K^trans^ and V_e_ between the DSI positive and DSI negative groups. **(C-E)** Boxplots showing the difference of APT_mean_, K^trans^, and V_e_ between the LVSI positive group and LVSI negative group.

Moreover, the APT_mean_, K^trans^, and V_e_ values in the LVSI positive group were significantly higher than those in the LVSI negative group (all *P*<0.05), while there was no significant difference in the K_ep_ value between the two groups (*P*>0.05). Details are shown in **Table 3** and **Figure 3**.

**Table 3:** Comparison of imaging parameters between DSI and LVSI positive and negative groups.

Parameters	Positive	Negative	*P*
	**DSI-positive (n=46)**	**DSI-negative (n=24)**	
AP_Tmean_	3.19 ± 0.63	2.91 ± 0.96	0.198
K^trans^ (min^-1^)	0.58 ± 0.23	0.37 ± 0.15	**<0.001**
K_ep_ (min^-1^)	0.89 ± 0.32	0.76 ± 0.29	0.104
V_e_	0.68 ± 0.28	0.52 ± 0.20	**0.018**
	**LVSI-positive (n=42)**	**LVSI-negative (n=28)**	
AP_Tmean_	3.35 ± 0.59	2.51 ± 0.70	**<0.001**
K^trans^ (min^-1^)	0.58 (0.42, 0.73)	0.34 (0.23, 0.51)	**<0.001**
K_ep_ (min^-1^)	0.89 ± 0.33	0.77 ± 0.29	0.119
V_e_	0.70 ± 0.30	0.51 ± 0.15	**0.002**

The bold values provided in Tables mean the differences were statistically significant.

### Logistic analysis of clinical and imaging parameters for predicting LVSI and DSI

K^trans^, V_e_ and SCC-Ag were included in binary logistic regression analysis, and K^trans^ and SCC-Ag were independent predictors to establish a prediction model for DSI: *Logti (P1) = -2.707 + (6.229×K^trans^) + (0.133×SCC-Ag).* APT_mean_, K^trans^, V_e_ and SCC-Ag were included in binary logistic regression analysis, and APT_mean_ and K^trans^ were independent predictors to establish a prediction model for LVSI: *Logti (P1) = -7.318 + (4.685×K^trans^) + (1.930×APT_mean_)*.

### The performance of significant parameters for predicting LVSI and DSI

**Figure 4:**
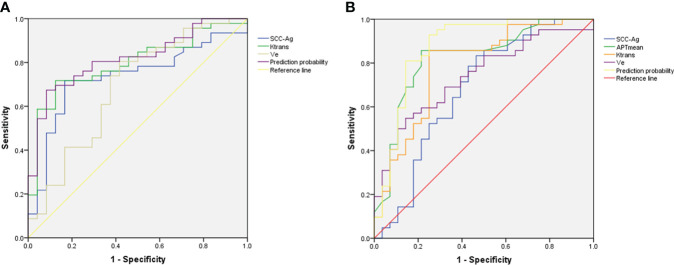
**(A)** ROC of K^trans^, V_e_, SCC-Ag and combined values (SCC-Ag+K^trans^) for the prediction of DSI. **(B)** ROC of SCC-Ag, APT_mean_, K^trans^, V_e_ and combined values (APT_mean_+K^trans^) for the prediction of LVSI. The combined values for DSI and LVSI both show the highest AUC.

The performance and ROC curves of SCC-Ag, K^trans^, V_e_ and the combined values (SCC-Ag+K^trans^) to predict DSI are shown in **Table 4** and **Figure 4A**, while the efficacy and ROC curve of SCC-Ag, APT_mean_, K^trans^, V_e_ and the combined values (APT_mean_+K^trans^) in predicting LVSI are shown in **Table 4** and **Figure 4B**.

**Table 4:** Diagnostic performances of significant parameters for predicting LVSI and DSI.

	AUC	Threshold value	Sensitivity	Specificity
**DSI**				
K^trans^	0.797	0.50	73.91%	87.50%
V_e_	0.689	0.50	80.43%	58.33%
SCC-Ag	0.733	2.71	71.74%	83.33%
SCC-Ag+K^trans^	0.819	0.73	71.74%	91.67%
**LVSI**				
SCC-Ag	0.680	1.69	83.33%	53.57%
APT_mean_	0.824	2.60	85.71%	78.57%
K^trans^	0.787	0.38	85.71%	75.00%
V_e_	0.739	0.66	59.92%	85.61%
APT_mean_+K^trans^	0.874	0.42	92.86%	75.00%

### Comparison of AUC between combined parameters and single parameters

The results of the Delong test showed that the AUC of K^trans^+SCC-Ag to predict DSI was not significantly different from that of K^trans^, SCC-Ag, or V_e_ alone (AUC_Ktran_ vs. AUC_Ktrans+SCC-Ag_, *P*= 0.489; AUC_SCC-Ag_ vs. AUC_Ktrans+SCC-Ag_, *P*= 0.186; AUC_Ve_ vs. AUC_Ktrans+SCC-Ag_, *P*= 0.061).

The AUC of APT_mean_+K^trans^ in predicting LVSI was higher than SCC-Ag alone (AUC_SCC-Ag_ vs. AUC_APTmean+Ktrans_, *P*= 0.025); however, there was no statistical difference between APT_mean_, K^trans^, and V_e_ alone and APT_mean_+K^trans^ AUC (AUC_APTmean_ vs. AUC_APTmean+Ktrans,_ *P*= 0.165; AUC_Ktrans_ vs. AUC_APTmean+Ktrans_, *P*= 0.056; AUC_Ve_ vs. AUC_APTmean+Ktrans_, *P*= 0.072).

### Comparison of clinicopathological parameters among the three groups

Patients without DSI or LVSI were classified into group 1, patients with only DSI or only LVSI were classified into group 2, those with co-occurrence of DSI and LVSI were in group 3.

The age was significantly different in group 1 vs. group 2 (*P*<0.05); the SCC-Ag value was different in group 1 vs. group 3 and group 2 vs. group 3 (both *P*<0.05), while group 1 vs. group 2 showed no statistical difference (*P*>0.05). There were no significant differences in FIGO stage, menstrual status, symptoms, histological type, and degree of differentiation among the three groups (all P>0.05). The results are shown in **Table 5**.

**Table 5:** Comparison of clinical characteristics, pathological features among the three groups.

Parameters	Value	P _1 vs. 2_	P_2 vs. 3_	P_1 vs. 3_
Age		**0.036**	0.141	0.847
Group 1 (n=11)*	49.6 ± 6.7			
Group 2 (n=30)*	58.4 ± 9.4			
Group 3 (n=29)*	53.3 ± 10.7			
FIGO stage (IB vs. IIA)		0.073	0.223	0.477
Group 1 (n=11)	5 vs. 6			
Group 2 (n=30)	23 vs. 7			
Group 3 (n=29)	18 vs. 11			
Menstruation (premenopausal vs. menopause)		0.064	0.297	0.293
Group 1 (n=11)	7 vs.4			
Group 2 (n=30)	8 vs.22			
Group 3 (n=29)	12 vs.17			
Symptom (vaginal bleeding vs. No vaginal bleeding)		0.361	0.731	0.660
Group 1 (n=11)	8 vs.3			
Group 2 (n=30)	26 vs.4			
Group 3 (n=29)	24 vs.5			
Histologic subtype (SCC vs. adenocarcinoma)		0.280	1.000	0.254
Group 1 (n=11)	6 vs. 5			
Group 2 (n=30)	22 vs. 8			
Group 3 (n=29)	22 vs. 7			
Differentiation degree (poor differentiation vs. well-moderate differentiation)		0.140	0.412	0.477
Group 1 (n=11)	5 vs. 5			
Group 2 (n=30)	15 vs. 6			
Group 3 (n=29)	15 vs. 8			
SCC-Ag		1.000	**0.003**	**0.002**
Group 1 (n=11)	1.27 (0.71, 2.71)			
Group 2 (n=30)	2.14 (0.98, 4.10)			
Group 3 (n=29)	4.82 (2.89, 6.95)			

*Group 1: DSI and LVSI were both negative; group 2: DSI or LVSI was positive; group 3: both DSI and LVSI were positive.

The bold values provided in Tables mean the differences were statistically significant.

### Comparison of imaging parameters among the three groups

The APT_mean_ in group 1 were significantly lower compared to group 2 (*P*=0.025), while there was no significant difference in K^trans^, K_ep_ and Ve values between group 1 and 2 (all *P*>0.05). Moreover, K^trans^ and V_e_ values in group 2 were significantly lower than those in group 3 (*P*<0.001 and *P*=0.010, respectively), while there was no significant difference in APT_mean_ and K_ep_ between the two groups (all *P*>0.05). The APT_mean_, K_trans_, and V_e_ values in group 1 were significantly lower than those in group 3 (*P*=0.001, *P*<0.001, *P*=0.002, respectively), while there was no difference in K_ep_. All data are shown in **Table 6**.

**Table 6:** Comparison of imaging parameters among the three groups.

Parameters	Value	P _1 vs. 2_	P_2 vs. 3_	P_1 vs. 3_
APT_mean_		**0.025**	0.287	**0.001**
Group 1 (n=11)*	2.29 ± 0.73			
Group 2 (n=30)*	2.93 ± 0.72			
Group 3 (n=29)*	3.22 ± 0.58			
K^trans^		0.219	**<0.001**	**<0.001**
Group 1 (n=11)	0.30 ± 0.12			
Group 2 (n=30)	0.41 ± 0.19			
Group 3 (n=29)	0.68 ± 0.18			
K_ep_		0.873	0.242	0.199
Group 1 (n=11)	0.74 ± 0.23			
Group 2 (n=30)	0.79 ± 0.32			
Group 3 (n=29)	0.95 ± 0.32			
V_e_		0.480	**0.010**	**0.002**
Group 1 (n=11)	0.45 ± 0.15			
Group 2 (n=30)	0.58 ± 0.18			
Group 3 (n=29)	0.77 ± 0.36			

*Group 1: DSI and LVSI were both negative; group 2: DSI or LVSI was positive; group 3: both DSI and LVSI were positive.

The bold values provided in Tables mean the differences were statistically significant.

## Discussion

This study combined clinical parameter SCC-Ag with DCE-MRI parameters and APT values to preoperatively predict the presence of DSI or/and LVSI in cervical cancer. The results demonstrated that SCC-Ag and K_trans_ were independent predictors of DSI, while APT_mean_ and K_trans_ were independent predictors of DSI. SCC-Ag, K_trans_ and Ve could discriminate the coexistence of DSI and LVSI from single risk factor, while APT_mean_ was able to discriminate the single risk factor from non-risk factors.

SCC-Ag is a specific antigen produced by squamous cell carcinoma and a common marker of cervical cancer. The higher SCC-Ag level indicates a higher malignancy. Previous studies have shown that DSI- or LVSI-positive patients have significantly higher SCC-Ag levels compared to DSI- or LVSI-negative patients (25, 26). In this study, the SCC-Ag was higher in the DSI- and LVSI-positive group compared to the corresponding negative group (*P*<0.05), which was similar to previous studies. However, the sensitivity of SCC-Ag in predicting DSI and the specificity in predicting LVSI were not yet satisfied.

As a non-invasive imaging technique reflecting microscopic tissue changes, APTw imaging has been applied to diagnose cervical cancer, central nervous system tumors, prostate cancer, endometrial cancer, and rectal cancer (19–21, 27–30). APT value is positively correlated with the degree of malignancy, i.e., the higher the degree of malignancy, the greater the APT value. In this study, the APT_mean_ of the LVSI positive group was significantly higher than that of the LVSI negative group. APT imaging reflects the content and concentration of macromolecular substances such as mobile proteins and polypeptides in organs and tissues; more intracellular protein/polypeptides and higher cell density have a greater APT value. Previous studies have found that the content of amide protons in tumor tissues is higher than that in normal tissues (31). Furthermore, Park et al. confirmed that the protein content in high-grade gliomas is higher than in low-grade gliomas (32). In addition, Liu and colleagues showed that the higher pathological tumor grade is associated with higher tumor cell density (33). LVSI-positive tumors are characterized by high aggressiveness. Compared to LVSI-negative tumor, cells of LVSI-positive tumor may associated with higher potential of malignant proliferation, and thus product and release more protein and polypeptide, that will lead to the higher APT_mean_ values.

DCE-MRI can assess tumor aggressiveness and treatment response by measuring the properties of tissue microvessels (34). This study showed that the K^trans^ values ​​of the DSI- and LVSI-positive groups were significantly higher compared to DSI- and LVSI-negative groups. Song et al. (35) and Li et al. (36) studied the relationship between parametrial invasion, a high-risk factor for cervical cancer, and DCE-MRI parameters, and found that the quantitative parameter values ​​of primary lesions in the infiltrating group were significantly higher than those in the non-infiltrating group. This was similar to the results of the present study. K^trans^ represents the rate at which the contrast agent in the tissue enters the extravascular extracellular space from the blood; the rate is affected by blood flow and permeability, e.g., higher K^trans^ indicate higher blood flow and permeability of the tissue. Aggressive malignant cells tend to proliferate rapidly, thus, a malignant tumor is often in a state of hypoxia, which stimulates tumor angiogenesis; while neovascular endothelial cells are immature and have high permeability (37). V_e_ reflects the extracellular space per unit volume of tissue. V_e_ value is positively correlated with larger extracellular space, might be more sensitive in detecting the enlarged space after local cell necrosis. In this study, the V_e_ of DSI- or/and LVSI-positive patients was higher than that of corresponding negative patients, which may imply more microscopic necrosis in DSI- and LVSI-positive tumors than that of negative tumors.

The results of multivariate analysis showed that Ktrans and SCC-Ag were independent factors of DSI, and APT_mean_ and K^trans^ were independent factors of LVSI. K^trans^ combined with SCC-Ag showed the largest AUC when predicting DSI. Compared with K^trans^, the sensitivity of the K^trans^+SCC-Ag value was lower, but its specificity reached 91.67%, which was higher compared to K^trans^ and SCC-Ag alone. As mentioned above, SCC-Ag is an important tumor marker for the diagnosis and evaluation of cervical cancer. This study shows that the combination of laboratory indicators and imaging parameters can further improve the evaluation value of single tumor biological behavior.

APT_mean_ combined with K^trans^ showed the largest AUC, with sensitivity and specificity of 92.86% and 75.00%, respectively, when predicting LVSI (the sensitivity was higher compared to K^trans^ or APT_mean_ alone (both 85.71%), while the specificity was the same as for K^trans^ (75.00%) and lower compared to APT_mean_ alone (78.57%)). Previous studies have suggested that increased microangiogenesis and increased blood perfusion in tumors lead to increased APT values (19). Therefore, we implied that LVSI-positive tumors have more neovascularization with immature blood vessel walls, which affects both APT value and K^trans^ in terms of blood perfusion and permeability so that APT_mean_ and K^trans^ have a synergistic effect on the identification of LVSI. Therefore, APT_mean_ combined with K^trans^ has a higher value in predicting LVSI. The AUC of APT_mean_ combined with K^trans^ was significantly higher compared to SCC-Ag, also revealed that the combination of APT and DCE-MRI could be more accurate than laboratory indicator alone in predicting LVSI.

In this study, patients were further divided into 3 groups: group 1: DSI and LVSI were both negative (11 cases); group 2, DSI or LVSI was positive (30 cases); group 3: both DSI and LVSI were positive (29 cases). For clinical characteristics, the age of group 2 was larger than the age of group 1, which indicated that younger patients tend to have no risk factors. However, age did not show the ability of predicting the coexistence of DSI and LVSI in this study. SCC-Ag can be used to discriminate group 2 from group 3, and group 1 from group 3, which also confirmed that SCC-Ag could be leveraged for assessing the malignant degree of cervical cancer and the risk factors. Imaging indices APT_mean_, K^trans^, V_e_ can all be used to discriminate the three groups of patients. K^trans^ and Ve can be used to discriminate group 2 from group 3, and group 1 from group 3, while only APT_mean_ can be used to discriminate group 1 from group 2. The invasiveness of the tumor with both DSI and LVSI (group 3) may be significantly stronger than in the other two groups. Also, APT_mean_, K^trans^, and V_e_ can all predict the coexistence of DSI and LVSI. It is speculated that group 2 may be less aggressive than group 3; in this study, it was more difficult to differentiate group 1 from group 2 than to differentiate group 1 from group 3. The APT_mean_ was more valuable in identifying patients without any DSI or LVSI.

This study has a few limitations. First, as a single-center retrospective study, the number of patients in this study is relatively small. Second, this study did not include rare histological tumor types such as small cell carcinoma. Third, this study only outlines the largest slice of the lesion. Although some previous studies used single-level ROI, considering the heterogeneity of tumors, further analysis of the entire tumor area should be carried out in the future. Also, radiomics may be used to compare image parameters and correlations. Associations with tumor biological behavior are better assessed.

## Conclusion

APT_mean_ and DCE-MRI quantitative parameters can help preoperatively predict the occurrence of DSI or/and LVSI. APT_mean_ and DCE-MRI parameters are able to identify the coexistence of DSI and LVSI, APT_mean_ may help clinicians identify the patients without any intermediate-risk factors before surgery. Therefore, APTw imaging combined with DCE-MRI have potential in the preoperative non-invasive prediction of intermediate-risk factors of cervical cancer.

## Data availability statement

The raw data supporting the conclusions of this article will be made available by the authors, without undue reservation.

## Ethics statement

The studies involving human participants were reviewed and approved by Institutional Review Board,First Affiliated Hospital of Dalian Medical University. Written informed consent for participation was not required for this study in accordance with the national legislation and the institutional requirements.

## Author contributions

QLS, AL, ST contributed to conception and design of the study. LC, XM collected clinical data. QLS, CM, measured MRI data. QWS, NW contributed to MRI data analysis and interpretation. QLS, LL, JW performed the statistical analysis. QLS finished draft of the manuscript, AL, ST contributed to supervision. All authors read and approved the final manuscript.

## Conflict of interest

Authors LL and JW were employed by Philips Healthcare.

The remaining authors declare that the research was conducted in the absence of any commercial or financial relationships that could be construed as a potential conflict of interest.

## Publisher’s note

All claims expressed in this article are solely those of the authors and do not necessarily represent those of their affiliated organizations, or those of the publisher, the editors and the reviewers. Any product that may be evaluated in this article, or claim that may be made by its manufacturer, is not guaranteed or endorsed by the publisher.

## Abbreviations

MRI, Magnetic resonance imaging; FIGO, International Federation of Gynecology and Obstetrics; SCC-Ag, squamous cell carcinoma antigen; APTw, Amide proton transfer weighted; CEST, Chemical exchange saturation transfer; DCE-MRI, Dynamic contrast-enhanced MRI; LVSI, Lymphovascular vascular space invasion; DSI, Deep stromal invasion; ROI, Regions of interest; ICC, Intra-group correlation coefficient; ROC, Receiver operating characteristic; AUC, Area under the curve.
