# Supplementary material for: Amide proton transfer weighted imaging combined with dynamic contrast-enhanced MRI in predicting lymphovascular space invasion and deep stromal invasion of IB1-IIA1 cervical cancer
Source: Front Oncol. 2022 Sep 12;12:916846. doi: 10.3389/fonc.2022.916846 (PMC9512406; doi:10.3389/fonc.2022.916846)
Supplement: Supplementary file 1 [file Table_1.docx]

**Table S1. The ICC of inter-observer reproducibility from two observers**

|  | Observer1 | Observer2 | ICC | 95% CI |
| --- | --- | --- | --- | --- |
| APT_mean_ | 2.93±0.71 | 2.94±0.73 | 0.904 | 0.858-0.926 |
| K^trans^ | 0.52±0.23 | 0.51±0.26 | 0.931 | 0.891-0.957 |
| K_ep_ | 0.86±0.35 | 0.87±0.35 | 0.924 | 0.881-0.952 |
| V_e_ | 0.65±0.32 | 0.63±0.28 | 0.873 | 0.803-0.919 |
